# Picture quiz

**Published:** 2014

**Figure:**
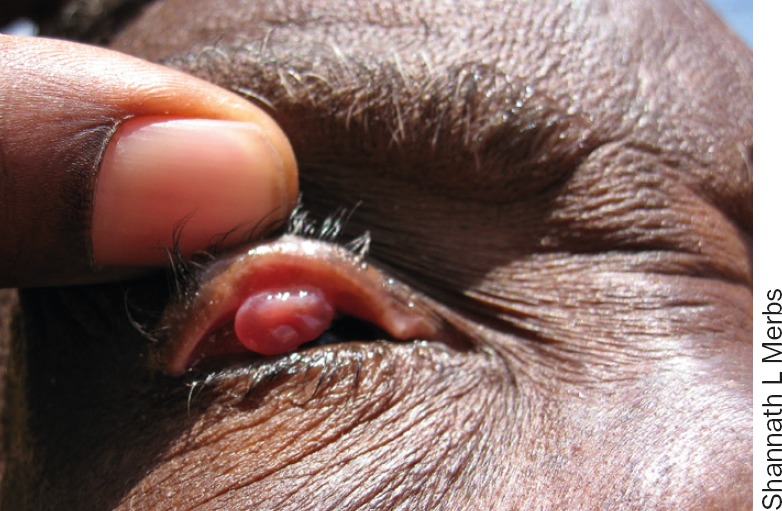


A 50 year-old woman in Africa presented with a history of eye pain and discharge for 3 months after upper eyelid surgery for trichiasis/entropion.

1. What is the diagnosis? (Select one.)
  □ **a.** Ptyergium of the conjunctiva
  □ **b.** Squamous cell carcinoma of the conjunctiva
  □ **c.** Granuloma of the conjunctiva
  □ **d.** Kaposi sarcoma of the conjunctiva
  □ **e.** Eyelid abscess
2. Which of the following are known risk factors for the answer to question 1? (Select all that apply.)
  □ **a.** Exposure to ultraviolet light
  □ **b.** HIV infection
  □ **c.** Retained suture fragment
  □ **d.** Male gender
  □ **e.** Malnutrition
3. Which of the following is the first line recommended treatment for the answer to question 1? (Select one.)
  □ **a.** Prednisolone drops
  □ **b.** Chloramphenicol ointment
  □ **c.** Cryotherapy
  □ **d.** Excision with a scalpel blade
  □ **e.** Radiotherapy

## ANSWER

1. Diagnosis: **c.** Granuloma of the conjunctiva following trichiasis surgery. This may occur 6 weeks to 6 months after the operation.
2. Risk factors: **c.** The commonest cause is retention of a fragment of non-absorbable suture left behind at the time of suture removal.
3. Treatment: **d.** Recommended treatment is local excision with a scalpel or scissors under topical anaesthesia.

